# Necrotizing crescentic glomerulonephritis related to sarcoidosis: a case report

**DOI:** 10.1186/s13256-015-0764-8

**Published:** 2015-12-14

**Authors:** Natallia Maroz, Halle Field

**Affiliations:** 1 Renal Physicians Inc., One Elizabeth Place, Suite 190, Dayton, OH 45418 USA; 2 Department of Medicine, Wright State University, Boonshoft School of Medicine, 128 E. Apple St., 2nd Floor, Dayton, OH 45409 USA; 3 Department of Medicine, Kettering Medical Center, 3535 Southern Blvd Kettering, Dayton, OH 45429 USA

**Keywords:** Crescentic glomerulonephritis, Rapidly progressive glomerular nephritis, Sarcoidosis

## Abstract

**Introduction:**

Renal injury due to sarcoidosis develops in less than a quarter of patients with this systemic disease. In most cases, granulomatous tissue alters the production of vitamin D, which leads to hypercalciuria, nephrocalcinosis, and nephrolithiasis. Granulomatous interstitial nephritis is another well-recognized pathological process associated with sarcoidosis. However, a glomerular pathology is very rarely noted, and only a few cases are reported to have cellular crescentic glomerulonephritis.

**Case presentation:**

We describe the case of a 26-year-old African American woman with systemic sarcoidosis, with a unique constellation of renal lesions, including noncaseating epithelioid granulomatous necrotizing interstitial nephritis, cellular crescent formation, and necrotizing vasculitis. Immunosuppressive therapy was helpful for alleviating her nephrotic syndrome and maintaining the stability of her renal function over a 30-month period.

**Conclusion:**

Glomerular involvement of sarcoidosis needs to be considered in the differential diagnosis in cases of rapidly progressive glomerular nephritis.

## Introduction

Sarcoidosis is a chronic systemic granulomatous disorder with various clinical presentations. Although lung involvement is the most common morbidity in such cases, severe extrapulmonary manifestations are also observed. Renal pathology may also be associated with sarcoidosis, and is observed in approximately 4–22 % of cases [1]. The spectrums of renal disorders include hypercalciuria, nephrocalcinosis, and nephrolithiasis, which are the most common causes of renal failure in sarcoidosis [2]. The second most-common manifestation of renal disease in sarcoidosis is granulomatous interstitial nephritis (GIN); its prevalence is reported to be 7–27 % [3]. Cases with glomerular lesions are rare, and only few cases have been reported to have evidence of crescentic glomerulonephritis [4]. In the present report, we describe a case wherein a patient with multisystem sarcoidosis exhibited a constellation of renal lesions, including GIN, crescentic glomerulonephritis, and necrotizing vasculitis.

**Fig. 1:**
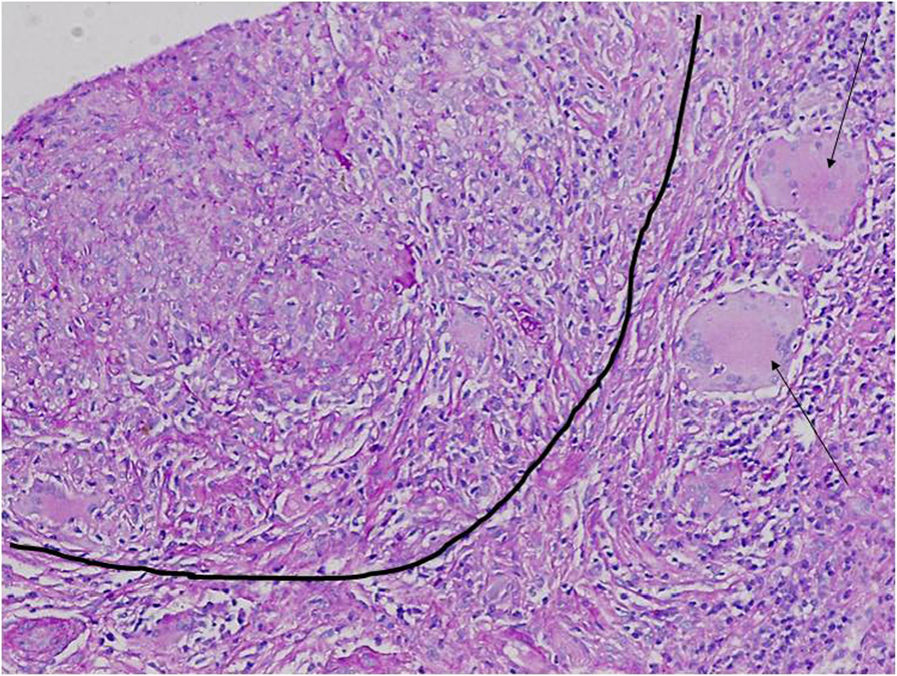
Granulomatous interstitial nephritis. Black line separates the zone of diffuse interstitial inflammation from the area with granulomatous responce. *Arrows* point toward epithelioid noncaseating granulomas (hematoxylin and eosin, 40X)

**Fig. 2:**
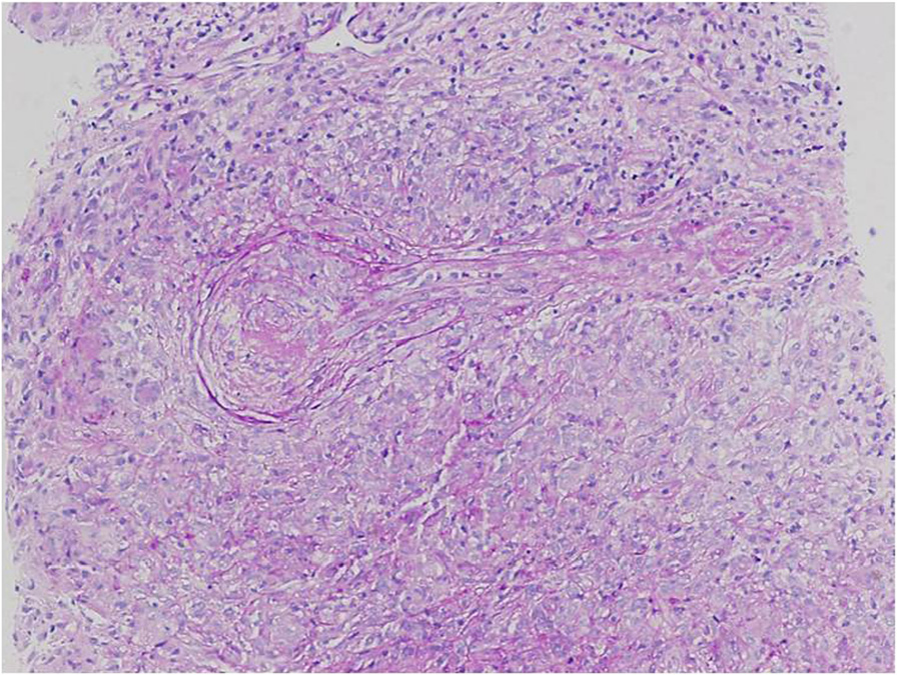
Necrotizing vasculitis in renal parenchyma (hematoxylin and eosin, 20X)

**Fig. 3:**
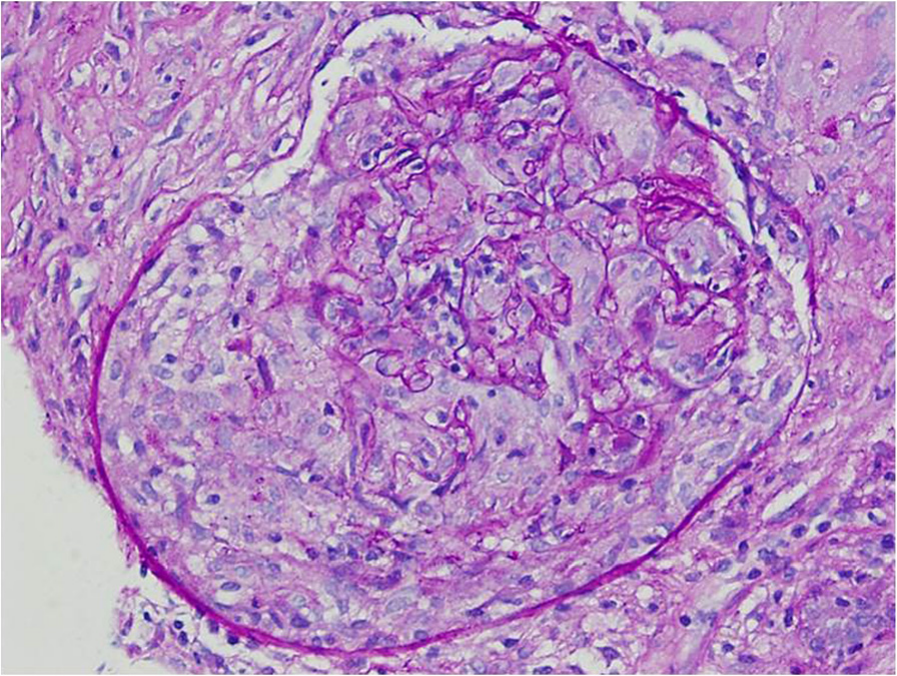
Cellular crescent formation (periodic acid-Schiff, 40X)

## Case presentation

A 26-year-old African American woman with accidental ankle subluxation presented to our emergency department with foot pain. As a part of her work-up, she was incidentally found to have an elevated serum creatinine level of 2.2 mg/dL and proteinuria. Therefore, our patient was referred to a nephrologist for further evaluation.

Her medical history included morbid obesity, hypertension, and hyperlipidemia. The hypertension had been present for 5 years, and was controlled with metoprolol (25 mg, twice a day) and amlodipine (10 mg, daily). Her other medications included ranitidine (150 mg, daily), lovastatin (20 mg, daily) and norgestimate ethinyl estradiol (one tablet, daily). Moreover, she took ibuprofen once a month, as needed, for shoulder pain. We did not note any personal or family history of kidney disease, autoimmune disease, or diabetes. The results of her physical examination were relatively unremarkable, except for obesity and moderate edema limited to her lower extremities. Her blood pressure was 140/90 mmHg. Examination of a 24-hour urine collection showed 11 g of urinary protein and hematuria. Her serum albumin level was 1.8 g/dL.

A kidney ultrasonography indicated normal size kidneys. Thereafter, she underwent a kidney biopsy, which showed severe noncaseating epithelioid granulomatous necrotizing interstitial nephritis(Fig. 1), necrotizing vascular lesion (Fig. 2), and cellular crescent formation (Fig. 3) that were related to the underlying sarcoidosis. Immunofluorescence studies were negative for immunoglobulin (Ig) IgA, IgM, kappa, lambda, C1q, C3, and fibrinogen but mild diffuse linear glomerular capillary staining for IgG was suggestive of pauci-immune crescentic glomerulonephritis.

Electron microscopy revealed no immune complex deposits. *Mycobacterium tuberculosis* studies indicated negative results. Serologic testing showed normal complement levels and negative results for anti-nuclear antibody, double-stranded DNA, antineutrophil cytoplasmic antibody (ANCA), human immunodeficiency virus, Sjogren’s syndrome, rapid plasma reagin, and viral hepatitis. Urine protein electrophoresis showed nonselective proteinuria related to combined glomerular and tubular damage. Her level of rheumatoid factor was <3 units/mL. Moreover, she was found to have elevated levels of angiotensin converting enzyme of 78 U/L (normal, 16–68U/L), but her levels of calcium, vitamin D, and parathyroid hormone were normal. A subsequent computed tomography (CT) scan of her chest showed patchy ground-glass opacities within both the lower lobes, with multifocal air trapping, as well as mild thickening of the bronchioles and mild hilar fullness, consistent with stage II pulmonary sarcoidosis. CT of her abdomen showed diffuse pelvic, para-aortic, and mesenteric lymphadenopathy. Examination of a pelvic lymph node biopsy specimen demonstrated no evidence of any lymphoproliferative disorder. Flow cytometry reported a predominance of T-cells in the tissue, with a normal 3.9:1 ratio of CD4 and CD8. Her lactate dehydrogenase level was within normal limits. Childhood records indicated a history of bilateral uveitis, which was treated with prednisolone.

Owing to the multisystem involvement, our patient was referred to a university-based sarcoidosis center. Because the renal pathology was predominant in her case, the management of immunosuppression was deferred to a nephrologist. She was initially treated with prednisone (60 mg, daily for 1 month), along with the subsequent addition of azathioprine (150 mg, daily). The prednisone administration was gradually tapered to 5 mg/day over 12 months. Subsequently, her nephrotic syndrome improved, her serum albumin levels improved to 3.8 g/dL, and her urinary protein levels markedly reduced from 11 g/24 hours to 1 g/24 hours. After 1 year, the concentration of interlukin-2 receptor in her peripheral blood was assessed, and the findings were suggestive of persistent disease activity. Hence, her azathioprine dose was increased to 200 mg/day.

Thirty months after diagnosis, our patient remains in partial renal remission, with stable stage 4 chronic kidney disease (creatinine, 2.5 mg/dL; glomerular filtration rate, 28 mL/min/1.73 m^2^) and minimal proteinuria.

## Discussion

Crescentic or rapidly progressive glomerular nephritis (RPGN) represents a nephrological emergency. It may be commonly linked to ANCA vasculitis, anti-glomerular basement membrane disease, or complement-mediated cases of lupus nephritis, cryoglobulinemia, IgA nephropathy, and post-infectious glomerulonephritis. The major clinical characteristics of the disease may include a marked decline in renal function along with active urinary sediment. Renal pathology demonstrates the presence of cellular crescents at the beginning of the disease, which frequently progress to fibrocellular crescents. If these patients are not promptly treated with immunosuppressive therapy, RPGN will lead to irreversible damage and end-stage renal disease [5].

Sarcoidosis is a chronic systemic granulomatous disorder with various clinical presentations and an unclear etiology. The pathological activity of T-cells appears to be an important event in the formation of noncaseating granulomas, although the triggering mechanism is unclear. Lung involvement is the most common morbidity, but extrapulmonary manifestations are also observed [6]. It is more prevalent in young women, and is often diagnosed around the age of 40 years. In the USA, this condition has a 3–10-fold greater incidence in African-American individuals than in Caucasians [2, 7]. At present, no universal diagnostic criteria have been developed for sarcoidosis; hence, diagnosis generally requires a biopsy and clinical exclusion of other granulomatous diseases [7].

The extrapulmonary manifestations of sarcoidosis affect the skin, eyes, nervous system, lymphatic system, joints, heart, and liver. Renal disease can also be associated with sarcoidosis, although such cases are less common and are observed in about 4–22 % of cases [1]. Moreover, hypercalciuria is noted in half of these patients as a result of calcitriol production in the granulomas. This further leads to nephrocalcinosis and nephrolithiasis, which are the most common causes of renal failure in sarcoidosis [2, 8]. The second most-commonly observed manifestation of renal disease in sarcoidosis is GIN; its prevalence is reported to be 7–27 % [3, 8]. In a retrospective analysis of 47 patients with sarcoidosis with renal involvement, 37 patients were reported to have noncaseating GIN. Granulomas with a lymphoma-like appearance may also be observed in renal sarcoidosis, and presents as a mass in the kidney parenchyma on imaging [8].

Glomerular lesions are very rarely observed, and include membranous nephropathy, focal segmental sclerosis, mesangioproliferative glomerulonephritis, IgA nephropathy, and crescentic glomerulonephritis [1, 9–11]. Among the cases of crescentic glomerulonephritis, half of the patients showed ANCA antibody positivity, raising concerns regarding the accuracy of the diagnosis and the similarity in the pathogenesis between two granulomatous diseases [8]. The presence of noncaseating necrotizing granulomas within the wall of small, medium, and even large vessels has been described in rare cases of systemic vasculitis from sarcoidosis [9, 10].

The presentation of renal involvement in sarcoidosis is unusual in our case. Our patient had a combination of GIN, RPGN, and necrotizing vasculitis in the absence of any other autoimmune disease. Moreover, she also presented with extensive extrarenal manifestations of sarcoidosis.

Sarcoidosis is not routinely considered to be a part of the differential diagnosis for RPGN or any other glomerular pathology; instead, it is diagnosed based on the exclusion of other possible conditions. Considerable literature is available to guide the management of RPGN not related to sarcoidosis, including the initial use of cytotoxic agents as induction therapy followed by the use of maintenance agents. It is unclear whether this treatment can be applied to the management of RPGN from sarcoidosis. Extrapulmonary manifestations of sarcoidosis are very responsive to prednisone therapy; however, successful use of cytotoxic agents has been reported in steroid-resistant cases [2, 12, 13, 14, 15]. Considering the severity of our patient’s renal manifestations and extrarenal disease, we started treatment with azathioprine along with prednisone. After 30 months, she showed partial renal remission and stabilization of other manifestations.

Although the therapeutic strategies for sarcoidosis-related RPGN need to be defined, a positive response to prednisone therapy is well recognized. A randomized multicentric clinical trial, “Treatment of renal sarcoidosis by methylprednisone bolus,” is currently recruiting patients to determine the required dose and duration of treatment with steroids. Use of adrenocorticotropic hormone (ACTH) gel appears to be an attractive alternative to corticosteroid therapy in extrarenal sarcoidosis via its effect on different subtypes of mineralocorticoid receptors (MCR). Positive renal effects were reported through the stimulation of MC1R in podocytes, reduced renal lymphocyte infiltration, effect on phospholipase A2 receptor, attenuation of glomerular immune complex deposition [16]. Different types of MCR have been identified in tubular epithelial cells and endothelial cells [17]; however, the validity of ACTH use in renal sarcoidosis should be confirmed through clinical evidence.

In the present case, our patient achieved partial renal remission with azathioprine and a low maintenance-dose of prednisone. Ideally, the use of prednisone-sparing agents is desired in order to avoid long-term corticosteroid toxicity. Studies regarding whether sarcoidosis can be managed with a prednisone-free regimen need to be conducted. Moreover, elucidating the etiology of sarcoidosis is essential to define the therapeutic aim and to develop targeted therapy.

## Conclusions

Glomerular involvement of sarcoidosis is a rare cause of RPGN. In certain cases, renal manifestations may precede the diagnosis of systemic sarcoidosis [5]. The management of sarcoidosis-related RPGN remains a clinical challenge. Therapeutic algorithms cannot be simply extrapolated from non-sarcoidosis types of RPGN. In the present case, we observed that the use of induction immunosuppressive therapy with cytotoxic agents was not required to achieve remission in a patient with sarcoidosis-related MPGN. Accumulating evidence via case reports of successful and unsuccessful strategies is important to achieve better outcomes in patients with sarcoidosis and RPGN.

## Consent

Written informed consent was obtained from the patient for the publication of this case report and accompanying images. A copy of the written consent is available for review by the Editor-in-Chief of this journal.
